# An Unusual Coexistence: Right-Sided Colon Cancer and Intestinal Malrotation in an Adult

**DOI:** 10.7759/cureus.55461

**Published:** 2024-03-03

**Authors:** Bruno Vieira, Artur Ribeiro, Paulo Sousa, Clara Leal, João Pinto-de-Sousa

**Affiliations:** 1 General Surgery Department, Centro Hospitalar de Trás-os-Montes e Alto Douro (CHTMAD), Vila Real, PRT

**Keywords:** gastrointestinal cancer, colon cancer, intestinal neoplasia, intestinal nonrotation, intestinal malrotation

## Abstract

Intestinal malrotation (IM), a rare congenital anomaly disrupting typical embryonic rotation around the superior mesenteric artery, is exceptionally uncommon in adults, with its link to colon cancer being even rarer. This article presents a case of colonic cancer in conjunction with IM in a 63-year-old male. Image studies and intraoperative findings show signs of IM. Open resection was performed due to concerns about vascular anomalies and abnormal lymphatic drainage. The case underscores the rarity of colon cancer in a malrotated gut, highlighting the necessity of preoperative identification for precise surgical planning and emphasizing the importance of careful dissection to prevent inadvertent vascular injury.

## Introduction

Intestinal malrotation (IM) is a congenital abnormality characterized by a deviation from the typical 270-degree counterclockwise rotation around the superior mesenteric artery (SMA) axis during embryonic development [1].

Its occurrence in adults is exceptionally low, at just 0.2%, and most symptomatic cases are typically identified shortly after birth [2]. It's unusual to find a link between IM in adults and right colon cancer, as evidenced by a few documented cases in the existing literature [2,3].

Achieving effective nodal clearance through surgical resection becomes challenging when confronted with abnormal anatomy and alterations in the vascular configuration in cases of IM.

The case we present involves a 63-year-old male patient who presented with right-sided colonic cancer. Upon examination, a growth was identified in the ascending colon, and he was treated via open right hemicolectomy. The infrequency of colon cancer in a malrotated gut, along with a meticulous perioperative evaluation, prompted us to share this case.

## Case presentation

A 63-year-old male, autonomous and independent, was referred to a colorectal appointment. He had a history of diabetes mellitus, for which he received antidiabetic medications, and there was no pertinent family medical history. He reported abdominal pain in the hypogastrium, which had been going on for about six months, anorexia for the same length of time, and weight loss of 5 kilograms in about 12 months. A colonoscopy was performed, uncovering a tumor in the cecum that was subsequently confirmed to be adenocarcinoma. The physical examination revealed no unusual observations.

Analytical tests demonstrated anemia, as indicated by a hemoglobin level of 11.1 g/dL (the normal range falls between 13.8 and 17.2 g/dL), with unaltered tumor markers, including carcinoma embryonic antigen (CEA) and carbohydrate antigen (CA).

**Figure 1:**
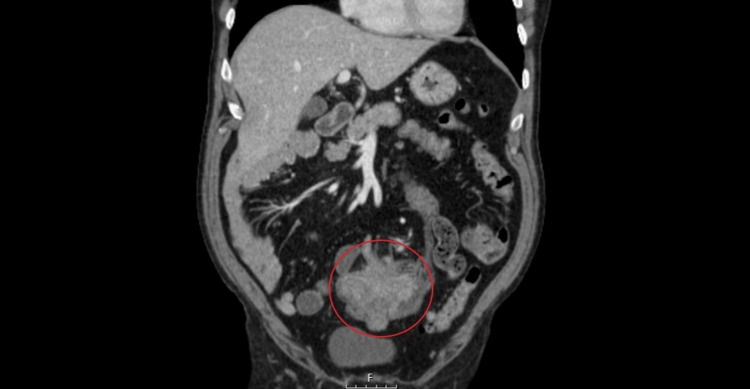
Coronal contrast-enhanced CT scan showing a caecum tumor located in the midline (red circle).

Contrast-enhanced computed tomography (CT) imaging of the chest, abdomen, and pelvis showed a 9x6 cm mass centered on the colonic lumen, extending into the pelvic region (Figure 1).

**Figure 2:**
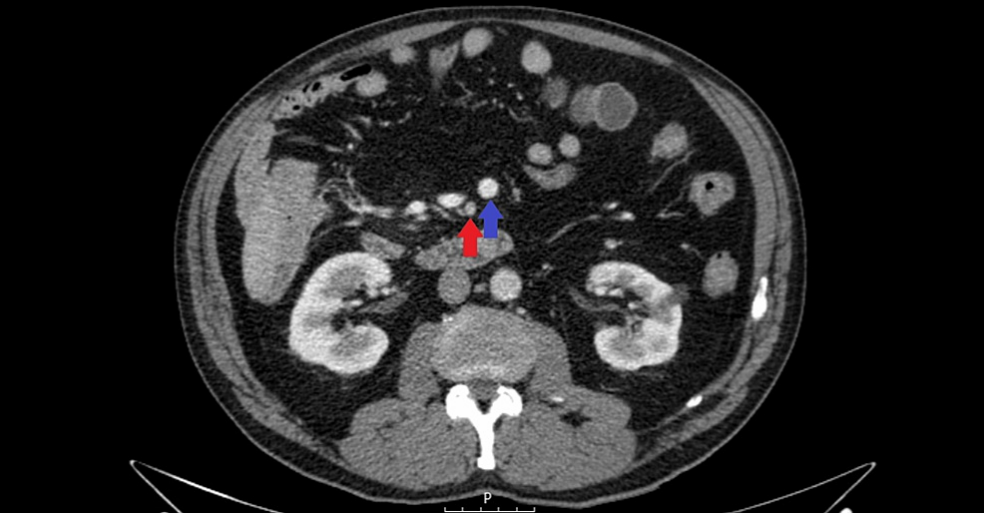
Axial contrast-enhanced CT scan showing an abnormal relationship between superior mesenteric vessels, with the SMA (red arrow) positioned to the right of the SMV (blue arrow). SMA: superior mesenteric artery, SMV: superior mesenteric vein

A concurrent 8 mm hypodense lesion was identified in the liver's segment VII. Due to imaging limitations, further characterization was recommended. Abdominal ultrasound did not yield conclusive results, prompting the use of magnetic resonance imaging (MRI) of the abdomen, which corroborated the hypothesis of its cystic nature. A staging CT showed no evidence of metastatic lesions; however, both this examination and the MRI revealed signs of IM. The imaging observations showed a mesenteric vessel arrangement in reverse, with the mesenteric artery notably on the right and the vein on the left (Figure 2).

**Figure 3:**
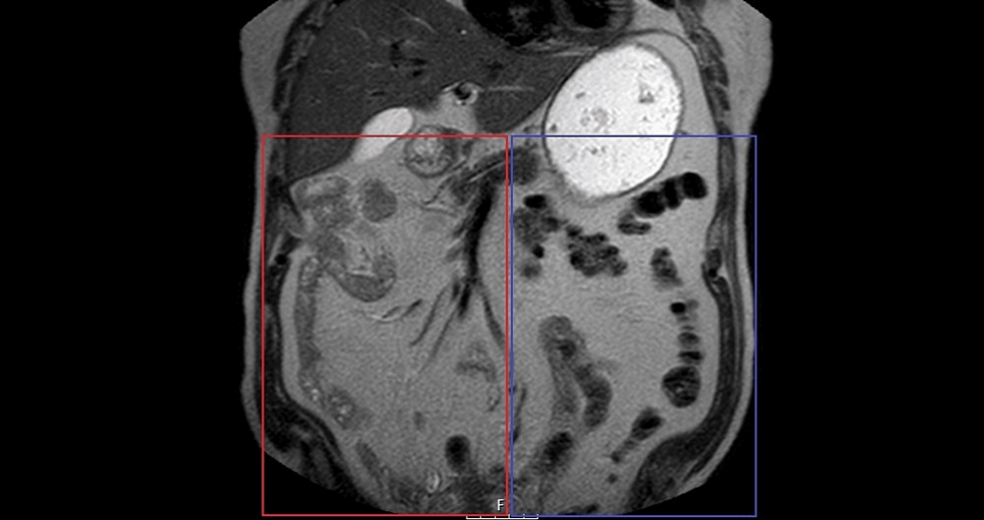
Coronal MRI showing the small intestine positioned on the right side of the abdominal compartment (red square), in contrast to the left-sided position of the colon (blue square).

Small bowel loops were consistently situated to the right of the midline, while colonic segments occupied the left quadrants, positioning the cecum and ileocecal valve anterior to the midline (Figure 3).

**Figure 4:**
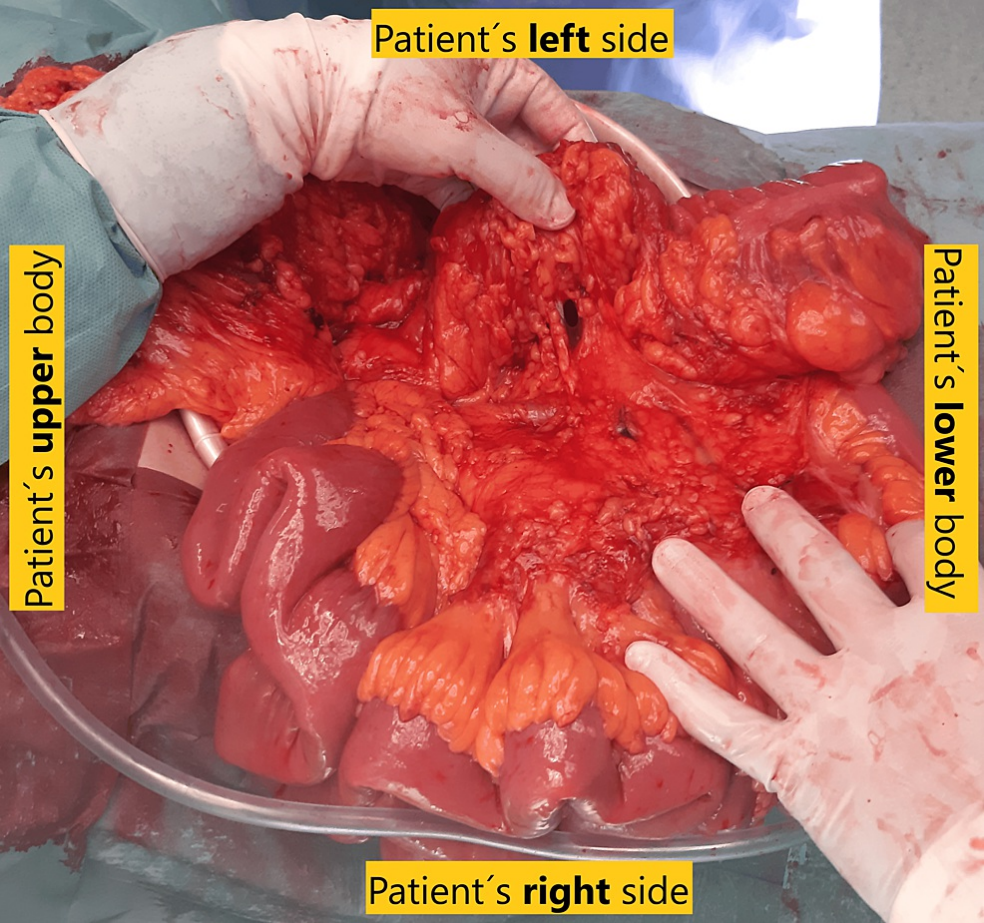
Intraoperative images during open right hemicolectomy showing the cecum positioned on the left side of the abdomen near the sigmoid colon and adhesions over the mesentery between the left and right colon.

Following discussions in a multidisciplinary tumor board meeting, surgical intervention was scheduled. The patient underwent an open right hemicolectomy with side-to-side anastomosis. Intraoperatively, examination revealed the cecum positioned on the left side of the abdomen near the sigmoid colon and the absence of fibrous peritoneal bands (Ladd’s bands) between the colon and the right abdominal wall (Figure 4). An abnormal relationship between superior mesenteric vessels was confirmed during surgery. Adhesions were identified over the mesentery between the left and right colon, requiring separation.

His recovery after surgery was uneventful, and he left the hospital on the fifth day postoperatively.

The histopathological analysis revealed mucinous adenocarcinoma in the cecum, staged as pT2 N0 (0/18) M0. The case was reviewed in a multidisciplinary tumor board meeting, and a decision was made for clinical surveillance. To date, there has been no evidence of disease progression or recurrence.

## Discussion

The swift asymmetrical expansion of the midgut initiates during the fifth week of gestation, involving herniation towards the proximal umbilical cord. A 270° counterclockwise rotation around the SMA occurs as the intestines re-enter the abdominal cavity by the 10th gestational week and secure attachment to the retroperitoneum. Developmental arrest at any point in this sequence results in malrotation [1,4].

There are different variations depending on the extent of rotation, broadly categorized as non-rotation, incomplete malrotation, and reverse rotation. Subtypes based on the orientation of the duodenum and colon further characterize these classifications [5]. When nonrotation occurs, there is a total absence of midgut rotation around the SMA. Consequently, the duodenojejunal segment is mainly located on the right side, and the large intestine is mostly confined to the left confined to the left quadrants of the abdominal compartment. In this case, there is a complete non-rotation of the intestine, where the entire midgut is affected, positioning the small bowel on the right side and the colon on the left side within the peritoneal cavity. A consistently observed characteristic of IM is the placement of the jejunum on the right side [6].

Malrotation of the gut associated with colon cancer appears to be more common in Japan compared to the global incidence. The majority of individuals diagnosed with colorectal cancer and IM are male, and the prevalence of cancer is higher on the right side [7].

In adults, malrotation is likely linked to gastrointestinal malignancies. There has been speculation about a potential link between malrotation and malignancy, drawing from reported cases, including our own. Studies have suggested that complications arising from right-sided colon cancer can result in chronic intestinal obstruction due to anatomical abnormalities in the colon, leading to inflammation and carcinogenesis [8]. Right colon cancer occurring in the context of IM is exceedingly uncommon, with only 39 documented cases worldwide [2,9,10].

An open resection was conducted due to worries about altered anatomy that could pose challenges to a safe resection and abnormal lymphatic drainage that might hinder a thorough lymphadenectomy. Several reports in the literature suggest that laparoscopic hemicolectomy is a safe and reliable approach for patients with right colon cancer and IM [2,10].

## Conclusions

Our images underscore the presence of colon cancer within a malrotated gut. Increasing awareness of the potential for malrotation may contribute to improved diagnostic accuracy in certain cases of colon cancer. The preoperative identification of malrotation enhances the precision of surgical planning. A meticulous dissection of the SMA and superior mesenteric vein (SMV) is crucial during surgery to prevent inadvertent injury. Further research is warranted to explore its potential association with malignancy.
